# Selegiline: a molecule with innovative potential

**DOI:** 10.1007/s00702-019-02082-0

**Published:** 2019-09-27

**Authors:** Tamás Tábi, László Vécsei, Moussa B. Youdim, Peter Riederer, Éva Szökő

**Affiliations:** 1 Department of Pharmacodynamics, Semmelweis University, Nagyvárad tér 4, 1089 Budapest, Hungary; 2 Department of Neurology, Interdisciplinary Excellence Centre, MTA-SZTE Neuroscience Research Group, University of Szeged, Faculty of Medicine, Albert Szent-Györgyi Clinical Center, Semmelweis u. 6, 6725 Szeged, Hungary; 3 Technion-Rappaport Family Faculty of Medicine, Efron St, PO Box 9697, 31096 Haifa, Israel; 4 Clinic and Polyclinic for Psychiatry, Psychosomatics and Psychotherapy, University Hospital Würzburg, Margarete-Höppel-Platz 1, 97080 Würzburg, Germany; 5 Department of Psychiatry, University of South Denmark, Odense, Denmark

**Keywords:** Selegiline, Monoamine oxidase type B inhibitors, Parkinson’s disease, Dopamine, Neuroprotection

## Abstract

Monoamine oxidase B (MAO-B) inhibitors have an established role in the treatment of Parkinson’s disease as monotherapy or adjuvant to levodopa. Two major recognitions were required for their introduction into this therapeutic field. The first was the elucidation of the novel pharmacological properties of selegiline as a selective MAO-B inhibitor by Knoll and Magyar and the original idea of Riederer and Youdim, supported by Birkmayer, to explore its effect in parkinsonian patients with on–off phases. In the 1960s, MAO inhibitors were mainly studied as potential antidepressants, but Birkmayer found that combined use of levodopa and various MAO inhibitors improved akinesia in Parkinson’s disease. However, the serious side effects of the first non-selective MAO inhibitors prevented their further use. Later studies demonstrated that MAO-B, mainly located in glial cells, is important for dopamine metabolism in the brain. Recently, cell and molecular studies revealed interesting properties of selegiline opening new possibilities for neuroprotective mechanisms and a disease-modifying effect of MAO-B inhibitors.

## History

The story of the monoamine oxidase B (MAO-B) inhibitors started with the discovery of selegiline (R-deprenyl). The compound was synthesized by Zoltán Ecseri in Chinoin Pharmaceuticals (Budapest, Hungary) in 1962 along with a series of similar, structurally related drug candidates. Their pharmacology was thoroughly studied in the laboratories of Joseph Knoll at Semmelweis University (Budapest, Hungary) (Knoll and Magyar 1972; Magyar et al. 1979; Knoll 1979). They were looking for psychic energizers based on the known antidepressant effect of MAO inhibitors (Knoll et al. 1965). The first clinical study was performed according to this intended indication (Varga and Tringer 1967). However, Knoll and Magyar observed peculiar features of deprenyl different from those of previously used MAO inhibitors, e.g., it lowers blood pressure and is free from “cheese effect”, the severe drug–food interaction of irreversible MAO inhibitor antidepressants. Namely, severe hypertensive crisis can develop when food rich in tyramine is consumed by patients treated by these drugs [for review see Finberg and Gillman (2011)]. The selective inhibitory effect of selegiline on one of the MAO isoforms was identified by Knoll and Magyar (1972). Parallel to their observation, Johnston published that the MAO enzyme exists in two distinct isoforms based on selective inhibition of one of them by clorgyline, which was named MAO-A (Johnston 1968). Selegiline, however, inhibits irreversibly the other isoform, and this was assigned as MAO-B. The other observation of Magyar was that the levorotatory isomer of the chiral deprenyl, R-deprenyl later called selegiline is a more potent inhibitor of MAO-B and has better pharmacological profile compared to the other deprenyl enantiomer (Magyar et al. 1967; Knoll and Magyar 1972).

The two MAO isoforms can be distinguished not only by their selective inhibitors, but they differ in their substrate specificity, as well. Serotonin and noradrenaline are specific substrates of MAO-A explaining the antidepressant and blood pressure increasing effects of its inhibitors (Finberg and Youdim 1983). β-Phenylethylamine was first identified as MAO-B substrate, since the selective inhibition of MAO-B increased the level of this biogenic amine (Reynolds et al. 1978). Only later on was it revealed that dopamine is also a substrate of MAO-B (Glover et al. 1977). Actually, it was shown to be a substrate of both MAO isoenzymes (O’Carroll et al. 1983).

It was the idea of one of us (P. R.) that an MAO inhibitor with favorable side effect profile was worth trying to treat Parkinson’s disease (PD) patients to prevent the serious on–off phenomenon. This suggestion was based on the clinical observations of Birkmayer and Hornykiewicz in the early 1960s that various MAO inhibitors provided mild clinical benefits in the treatment of PD (Birkmayer and Hornykiewicz 1962). However, the potential hypertensive effect of the non-selective irreversible MAO inhibitors restricted their further use. M. Y. informed P. R. about the new selective MAO-B inhibitor compound, selegiline, free from the “cheese effect” and without liver toxicity, which is another problem with some irreversible MAO inhibitors. Although at that time they were not aware that dopamine was a good substrate of MAO-B, furthermore based on rodent studies it was rather regarded as a MAO-A substrate, after some debate P. R., M. Y. and Birkmayer finally concluded that selegiline was worth trying in PD. M. Y. had got some selegiline from Knoll for experimental works and he provided it for starting to treat parkinsonian patients in 1974. The benefit of the treatment with the combination of levodopa and selegiline was first published in 1975 (Birkmayer et al. 1975) and 2 years later its long-term effectiveness was also reported (Birkmayer et al. 1977).

Later on, human post-mortem brain studies revealed that MAO-B isoform was present in the human brain, predominantly in glia (Konradi et al. 1989; Riederer et al. 1987; Collins et al. 1970). In various regions of post-mortem brain of selegiline-treated patients, elevated β-phenylethylamine and dopamine levels were detected (Riederer et al. 1984; Riederer and Youdim 1986), supporting the dopamine-sparing effect of the MAO-B inhibition, which is in line with the clinical findings. Platelet MAO-B inhibition in selegiline-treated patients was also demonstrated (Riederer et al. 1978).

On the proposal of two of us (P. R. and M. Y.) data from PD patients treated for a long time with selegiline in the Lainz Geriatric Hospital, Vienna, were analyzed. The interesting results demonstrated a prolongation of life expectancy for selegiline-treated PD patients first published in 1983 (Birkmayer 1983). The re-analysis of the data by more sophisticated statistical methods showed the same result (Birkmayer et al. 1985). These publications gave a new impetus on preclinical and clinical studies on selegiline aiming at elucidation of its possible neuroprotective effect.

Experimental data from numerous animal and cell culture studies provided evidence that selegiline protects against various neurotoxins, reduces oxidative stress and possesses neurotrophic and antiapoptotic effects. All these properties may contribute to its neuroprotective activity. These preclinical findings were previously thoroughly reviewed (Gerlach et al. 1996; Magyar et al. 2004, 2006; Naoi and Maruyama 2010; Maruyama and Naoi 2013; Szoko et al. 2018).

## Clinical studies on selegiline in PD

The increasing amount of preclinical pharmacological data about the possible neuroprotective effect of selegiline raised the hypothesis that it may slow the progression of PD. To prove this concept, several placebo-controlled randomized clinical trials were initiated from the late 1980s. A smaller study of 54 patients published in 1989 was the first reporting that 10 mg/day selegiline treatment delayed the need for levodopa therapy by about 8 months, which was ascribed to a possible disease progression-slowing effect (Tetrud and Langston 1989). However, it was immediately debated because the bare symptomatic effect of selegiline resulting from its dopamine-sparing effect could not be ruled out (Friedhoff 1990). The first large-scale multicenter controlled clinical trial was initiated in 1987 and it also aimed at determining the disease-modifying effect of selegiline in early PD, which was supposed to be related to the recognized antioxidant property of MAO inhibition. Hydrogen peroxide is one of the products of MAO-catalyzed amine oxidation reaction and it can be converted to further reactive oxygen species resulting in cytotoxicity. The inhibition of MAO activity thus may reduce oxidative stress. As the putative mechanism of neuroprotection was the antioxidant property of selegiline, another antioxidant, tocopherol, was also included in the study in addition to placebo as comparator. Eight hundred untreated PD patients were enrolled into this DATATOP study with primary end point of development of disability, necessitating the introduction of levodopa treatment. In the interim reports evaluating the first 12 months of the trial, a 57% reduction in the number of subjects reaching the primary end point was shown (Parkinson Study Group 1989) and 50% less patients had to give up full-time employment (Shoulson 1992) in the 10 mg/day selegiline-treated group. The final report was based on the results of a mean 14 ± 6 months treatment period. Selegiline therapy delayed the time to end point, the need of levodopa treatment, by about 9 months while tocopherol was ineffective. The mechanism of this beneficial effect was not revealed in the study, but the importance of the antioxidant property was questioned. Regarding symptoms control, assessed by Unified PD Rating Scale (UPDRS) and Activity of Daily Living (ADL) scores, selegiline was most effective in the first 3 months of the therapy. However, 2 months after its withdrawal, the motor performance of patients declined (Parkinson Study Group 1993). These findings clearly indicate the symptomatic benefit of selegiline treatment, but its disease-modifying effect was not unequivocally proven. In a continuation of the study, selegiline was administered to all patients who did not reach the primary end point over 21 ± 4 months of observation. There was no significant difference between the previously selegiline- or placebo-treated groups in the time reaching the end point, the need of starting levodopa. The conclusion of the Parkinson Study Group was that the initial benefit of selegiline treatment was not sustained (Parkinson Study Group 1996). In another continuation, those selegiline-treated patients who required levodopa were re-randomized after 5 years to continue selegiline or change to placebo. During the 2 years follow-up, there was no significant difference in combined end points of development of wearing-off, dyskinesia and on–off fluctuation between the treatment groups. However, assessing the distinct motor disturbances, there were less freezing of gait, wearing-off and on–off fluctuation, but more dyskinesia in the selegiline group, which is consistent with its dopamine-potentiating effect (Shoulson et al. 2002). Further smaller studies were also performed to evaluate the advantage of selegiline monotherapy in early PD. Rapid improvement in several parkinsonian symptoms was demonstrated in several short-term studies (Allain et al. 1993; Mally et al. 1995). Some other trials, similarly to DATATOP study, aimed at evaluation of disease-modifying effect of selegiline. In the Finnish study 54 untreated patients were randomized to 10 mg/day selegiline or placebo. The median time to initiation of levodopa treatment was found about 6 months longer (Myllyla et al. 1991) and the necessary dose of levodopa for the sufficient therapeutic effect was about half in the selegiline group (Myllyla et al. 1993). The levodopa dose-sparing effect was not only maintained, but further increased after the 5-year follow-up period. Selegiline treatment also reduced the need of additional dopaminergic therapy (slow release levodopa or dopamine agonist) (Myllyla et al. 1997). In a Swedish study of 157 early PD patients, selegiline significantly delayed the need to start levodopa therapy by about 4 months compared to placebo. Furthermore, the advantage of selegiline treatment in UPDRS scores was maintained after 2 months washout period before levodopa was started suggesting its neuroprotective effect (Palhagen et al. 1998). In the continuation of this study, the advantage of selegiline was also maintained after levodopa was started. Patients after 5 years on selegiline and levodopa combination had nearly ten points lower UPDRS scores, while 19% lower levodopa dose was used compared to levodopa only group (Palhagen et al. 2006). Recently, the benefit of selegiline monotherapy in early PD was also confirmed in a 12-week controlled trial in Japan, further supporting the use of the drug in early Parkinsonism (Mizuno et al. 2017).

These clinical trials unequivocally proved the efficacy of selegiline monotherapy in early PD (Table 1). In addition to symptoms improvement, it delays the introduction of levodopa by 3–9 months and reduces the necessary levodopa dose. Some of the studies also indicate the long-term maintenance of the initial benefit, suggesting neuroprotective effect of early started selegiline treatment.

**Table 1:** Clinical trials evaluating the effect of selegiline monotherapy in early PD

Design, number of patients (study name)	Studied daily dose	Key findings	References
Double-blind RCT, 54 patients	10 mg selegiline vs. placebo	8 months delay in the need of initiation of levodopa therapy	Tetrud and Langston (1989)
Double-blind RCT, 800 patients (DATATOP—Interim report)	10 mg selegiline vs. placebo	57% reduction in the number of patients needing levodopa after 12 months treatment	Parkinson Study Group (1989), Shoulson (1992)
Double-blind RCT, 54 patients	10 mg selegiline vs. placebo	6 months delay in the need of initiation of levodopa therapy. About half dose levodopa was needed in the selegiline group	Myllyla et al. (1991) Myllyla et al. (1993)
Double-blind RCT, 93 patients	10 mg selegiline vs. placebo	Improved motor rating and depressive scores after 3 months in the selegiline group	Allain et al. (1993)
Double-blind RCT, 800 patients (DATATOP—final report)	10 mg selegiline vs. placebo	9 months delay in the need of initiation of levodopa therapy	Parkinson Study Group (1993)
Double-blind RCT, 20 patients	10 mg selegiline vs. placebo	Improved motor scores after 3 weeks in the selegiline group	Mally et al. (1995)
Open label continuation of DATATOP, 310 patients	10 mg selegiline	No difference in benefit of early and late start of selegiline	Parkinson Study Group (1996)
Double-blind RCT, 157 patients	10 mg selegiline vs. placebo	4 months delay in the need of initiation of levodopa therapy. Improved motor scores were maintained after 2-month washout	Palhagen et al. (1998)
Double-blind RCT, 292 patients	10 mg selegiline vs. placebo (dose was escalated over 6 weeks)	Improved motor scores after 12 weeks in the selegiline group	Mizuno et al. (2017)

In addition to the continuations of monotherapy trials, combinational therapy studies were performed, as well. In the first double-blind randomized trial, 112 patients poorly responding to levodopa were subjected to 7.5 mg/day selegiline or placebo combination therapy. After 8 weeks, significantly more patients showed moderate or better improvement when selegiline was added to levodopa (Takahashi et al. 1994). Olanow et al. compared the deterioration of early PD patients receiving levodopa/carbidopa with or without selegiline combination for 12 months, followed by 2 months selegiline washout period. The UPDRS score was deteriorated by 5.8 ± 1.4 points in the levodopa/carbidopa group, but remained unaltered (0.4 ± 1.3 point deterioration) in the levodopa/carbidopa + selegiline group. Because of the 2 months selegiline washout before the evaluation, these data suggest its disease-modifying rather than barely symptomatic effect (Olanow et al. 1995). Later on, the study design was criticized because there were no placebo and selegiline only groups. However, according to the authors’ answer, for demonstration of benefit of add-on selegiline treatment, the presented design was appropriate (Schulzer 1997). The long-term benefit of addition of selegiline to levodopa treatment was reported by the Norwegian–Danish study group, as well. In the randomized placebo-controlled double-blind trial, combination treatment was continued for 5 years followed by 1-month selegiline washout. At the end of the study, symptoms were less severe and levodopa dose was lower when selegiline was combined to levodopa/benserazide and no symptom worsening was observed after the washout period (Larsen et al. 1999). SELEDO trial evaluated the long-term outcome of early started combination treatment of levodopa with selegiline or placebo in 160 patients. The primary end point was the need of more than 50% increase in levodopa dose for symptoms control. Almost twice longer time (4.9 vs. 2.6 years) was needed to reach the end point in the selegiline combination group (Przuntek et al. 1999).

In addition to prospective studies, retrospective analysis of patient registers was also performed with contradictory results in terms of effect on disease progression. In one of the early reports, no significant difference between selegiline + levodopa and levodopa alone groups was found regarding Hoehn–Yahr stage and motor symptoms in a period of 5 years. The levodopa dose, however, was lower in case of the combination treatment (Brannan and Yahr 1995). The benefit of long-term and/or early started selegiline treatment is indicated by other retrospective data analyses. Mizuno et al. reported that patients on combination of levodopa and selegiline had lower UPDRS scores compared to levodopa only treated group after about 10 years of disease duration. Adding selegiline to levodopa at this time point, the UPDRS score, although improved after the 4-month combination treatment, did not reach the level seen in patients on early started combination therapy (Mizuno et al. 2010). Another study evaluated the factors affecting disease progression in 687 patients assessing the retardation of Hoehn–Yahr stage transition time. It was found that in early PD, at least 3-year selegiline treatment increased the transition times from stage 2 to 2.5 and 2.5 to 3 (Zhao et al. 2011). Table 2 summarizes the results of studies on combinational therapies.

**Table 2:** Clinical trials and retrospective studies evaluating the effect of selegiline combinational therapy in PD

Design, number of patients (study name)	Studied daily dose	Key findings	References
Double-blind RCT, 112 patients	7.5 mg selegiline vs. placebo in combination with levodopa	More improvement after 8 weeks in the selegiline group	Takahashi et al. (1994)
Retrospective, 82 patients	10 mg selegiline in combination with levodopa vs. levodopa alone	No difference in motor scores after 1–5 years Lower levodopa dose needed after 1–3 years in the selegiline group, no difference after 4–5 years	Brannan and Yahr (1995)
Double-blind RCT, 101 patients	10 mg selegiline vs. placebo in combination with levodopa and/or bromocriptine	Less disease deterioration after 1 year in the selegiline group. Benefit was maintained after 2-month washout	Olanow et al. (1995)
Double-blind continuation of Myllyla’s study, 44 patients	10 mg selegiline vs. placebo in combination to levodopa	Lower levodopa dose needed even after 5 years in the selegiline group	Myllyla et al. (1997)
Double-blind RCT, 163 patients	10 mg selegiline vs. placebo in combination with levodopa	Less disease deterioration after 5 years in the selegiline group. Benefit was maintained after 1-month washout	Larsen et al. (1999)
Double-blind RCT, 160 patients	10 mg selegiline vs. placebo in combination with levodopa	About twice longer time to need for 50% increase in levodopa dose in the selegiline group	Przuntek et al. (1999)
Double-blind continuation of DATATOP, 368 patients	10 mg selegiline vs. placebo in combination with levodopa	Less wearing-off, freezing of gait and on–off fluctuation and more dyskinesia in the selegiline group	Shoulson et al. (2002)
Double-blind continuation of Palhagen’s study, 140 patients	10 mg selegiline vs. placebo in combination with levodopa	Improved motor scores with 19% lower levodopa dose after 5 years in the selegiline group	Palhagen et al. (2006)
Retrospective and open label, 691 patients	10 mg selegiline in combination with levodopa started within 5 years of disease onset vs. levodopa alone. 4-month open label selegiline treatment initiated in the levodopa alone group	Improved motor scores after 10 years in the selegiline group. Motor scores were improved after 4-month selegiline addition, the late started selegiline was less effective than the early started one	Mizuno et al. (2010)
Retrospective, 687 patients	Effect on disease progression of various pharmacotherapies with or without selegiline was evaluated	At least 3 years selegiline treatment of early PD patients increased the time to progression (Hoehn–Yahr stage transition)	Zhao et al. (2011)

Contrary to the increased life expectancy of selegiline-treated PD patients in the early observations of Birkmayer (1983) and Birkmayer et al. (1985), the Parkinson’s Disease Research Group of the UK reported increased mortality of patients treated with selegiline in combination with levodopa compared to levodopa alone during 5–7 years of follow-up (Lees 1995; Ben-Shlomo et al. 1998). However, meta-analyses of the available clinical trials with selegiline or any MAO-B inhibitors found no increase in mortality (Olanow et al. 1998; Ives et al. 2004; Macleod et al. 2005).

Based on the favorable effects of selegiline in PD, another irreversible MAO-B inhibitor, rasagiline, was introduced into the therapy. It was patented in 1995 (Youdim et al. 1995). Interestingly its methylated derivative, J-508, has already been tested in Knoll’s laboratory for MAO inhibition in the 1970s and was found to be slightly more potent in vivo compared to selegiline (Knoll et al. 1978). The pharmacological properties of rasagiline have been described by one of us (M. Y.). Its MAO inhibitory potential and selectivity in vitro are similar to those of selegiline, but in vivo it is about five times more potent probably due to pharmacokinetic differences (Youdim et al. 2001). A plethora of further preclinical studies showed rasagiline possessing a similar profile of beneficial effects as selegiline (Weinreb et al. 2010, 2011).

The effectiveness of rasagiline was evaluated in large clinical trials. In the first TEMPO study, its rapid symptomatic effect in early PD was demonstrated (Parkinson Study Group 2002). The results of the open-label extension of this trial indicated that patients on early started rasagiline treatment had better UPDRS score compared to delayed rasagiline administration during 6.5 years of follow-up, suggesting its neuroprotective property (Hauser et al. 2009). These results are similar to those found in selegiline studies. The ADAGIO study was designed to compare the effect of early vs. delayed start of rasagiline in controlled, randomized trial as well, to explore its disease-modifying property. The results, however, are contradictory because the extra benefit of early started therapy was observed only in case of the lower (1 mg/day) dose (Olanow et al. 2009). The effectiveness of rasagiline in levodopa-treated patients was also proved in the PRESTO and LARGO studies (Parkinson Study Group 2005; Rascol et al. 2005; Elmer 2013).

There are some further recently published data on the neuroprotective effect of the MAO-B inhibitors in PD. During 2007–2013, a large clinical study, NET-PD LS1, was performed, where the primary goal was to evaluate the effect of dietary supplement creatine on the progression of PD. Analysis of data of 1616 participants with mean observation time of 4.1 years revealed that 784 patients receiving MAO-B inhibitor (either selegiline or rasagiline) showed less clinical decline and this benefit was proportional to the cumulative duration of MAO-B inhibitor use. The effect of selegiline and rasagiline was not analyzed separately. These results indicate that MAO-B inhibitors may slow the disease progression (Hauser et al. 2017). A recent meta-analysis of the available clinical data confirms the effectiveness of MAO-B inhibitors both in monotherapy and in combination with levodopa. According to its results, selegiline seems to be the most effective MAO-B inhibitor in the combination therapy (Binde et al. 2018).

There are several recent reviews discussing the place of MAO-B inhibitors in the therapy of PD (Riederer and Laux 2011; Fabbrini et al. 2012; Dezsi and Vecsei 2014; Marsili et al. 2017; Dezsi and Vecsei 2017; Riederer and Muller 2018). More and more data suggest the disease-modifying effect of selegiline or other MAO-B inhibitor therapy, especially when they are started early and used long term (Muller and Mohr 2019). However, because of their symptoms-improving effect, the mechanisms behind the reported clinical benefits could not be unequivocally distinguished so far (Olanow 2009; Teo and Ho 2013).

## Selegiline in Alzheimer’s disease

Based on its widely examined neuroprotective property, selegiline was also studied as a potential therapeutic tool for Alzheimer’s disease. Some smaller early trials indicated benefit on both cognitive and behavioral symptoms after 3–6 months of treatment. Good tolerability of the drug was also confirmed in these studies (Campi et al. 1990; Monteverde et al. 1990; Mangoni et al. 1991; Finali et al. 1992; Filip and Kolibas 1999). However, there were some trials that showed only slight, clinically not relevant improvements in cognitive and/or behavioral functions (Tariot et al. 1987; Burke et al. 1993). The conclusion of the meta-analyses is that selegiline may provide mild short-term benefit, but its magnitude is not clinically meaningful (Wilcock et al. 2002; Birks and Flicker 2003).

The mild benefit in Alzheimer’s disease and the neuroprotective activity shown in preclinical experiments initiated the development of new drug candidates including multitarget compounds. Molecules containing MAO and cholinesterase inhibitory pharmacophores were designed and found having neuroprotective activities on various in vitro and in vivo animal models (Weinreb et al. 2012; Unzeta et al. 2016). However, no clinical data with these compounds have been reported so far.

## Delivery forms of selegiline

Based on the early clinical data of Birkmayer’s studies, selegiline was first licensed in Hungary in 1977 by Chinoin Pharmaceuticals under the brand name of Jumex^®^. At that time, it was recommended for alleviation of on–off phenomenon in levodopa-treated PD patients. Some years later, it was also approved in the same therapeutic indication in UK (1982) and in the USA as an orphan drug in 1989.

After the previously discussed clinical trials, the use of selegiline tablets was extended to early monotherapy of PD in the European Union in 1993 and in the USA in 1997. Since then the drug has been marketed worldwide.

**Fig. 1:**
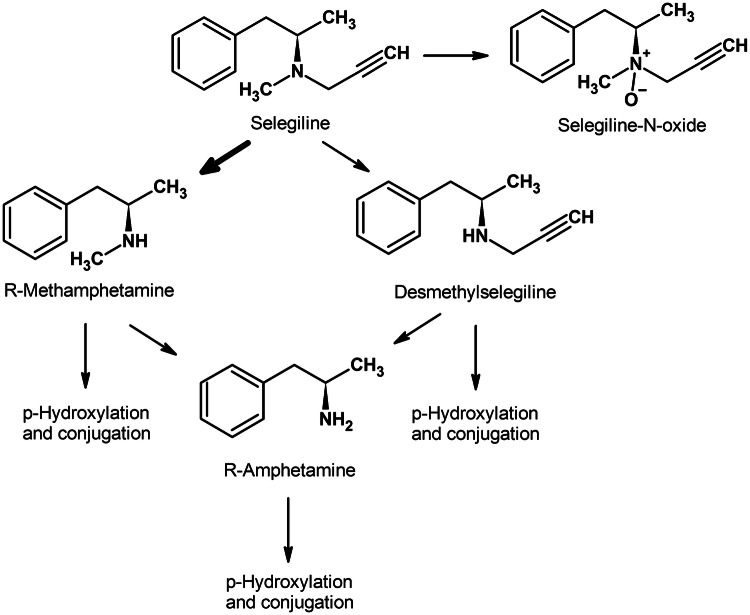
Scheme of metabolic transformation of selegiline. The thick arrow indicates the main metabolic step (the formation of R-methamphetamine)

After oral administration, selegiline undergoes extensive first pass metabolism and systemic exposure of its metabolites is considerably higher than that of the parent compound. R-Methamphetamine is its primary metabolite that is partially converted to R-amphetamine. In low quantity, desmethylselegiline and selegiline-*N*-oxide are also formed as shown in Fig. 1 (Heinonen et al. 1989; Szoko et al. 1999; Tabi et al. 2003; Shin 1997). These metabolites are pharmacologically active and their contribution to dopaminergic and neuroprotective effects of selegiline was suggested on the basis of extensive preclinical experiments (Szende et al. 2001; Magyar et al. 2004, 2006, 2010). However, Sandler et al. reported that substitution of selegiline treatment by its metabolites (mixture of R-methamphetamine and R-amphetamine) resulted in loss of clinical effect, suggesting no contribution of amphetamines to the clinical benefit in Parkinson’s disease (Elsworth et al. 1982; Stern et al. 1983). In clinical trials, selegiline was found to be well tolerated with similar side effect profile to that of placebo and no amphetamine-related adverse events were observed (Shoulson 1992; Palhagen et al. 1998; Shoulson et al. 2002; Palhagen et al. 2006; Mizuno et al. 2017). The weaker psychotropic effect and dependence liability of R-amphetamines compared to the S-enantiomers were shown in several preclinical studies supporting the findings of the clinical reports (Nickel et al. 1994; Yasar et al. 1993).

To improve its bioavailability, new delivery forms of selegiline were also developed. Orally disintegrating tablets (ODT) are characterized by rapid drug release into saliva. A fraction of the active pharmaceutical ingredient absorbs through the buccal mucosa, avoiding thus the first pass metabolism and providing fast onset of action. According to comparative pharmacokinetic studies of conventional and ODT formulations, selegiline plasma exposure of 1.25 mg ODT was comparable to that of 10 mg conventional tablet. Plasma levels of metabolites were considerably lower in case of the ODT preparation (Clarke et al. 2003a). Comparative clinical trial of 1.25–2.5 mg selegiline ODT and 10 mg conventional tablets revealed similar efficacy and safety (Clarke et al. 2003b). Based on this and some further clinical data, selegiline ODT was approved by FDA in 2006 and EMA in 2010 as an adjunctive therapy to levodopa in PD. By reviewing the available clinical data, we concluded that the clinical advantage of the clear pharmacokinetic improvement is not well justified. However, the patients’ preference, because of the convenience of ODT use, especially in patients with swallowing difficulties, should be acknowledged (Tabi et al. 2013).

Another innovative formulation, selegiline transdermal system (STS), was developed to avoid first pass metabolism. Using STS, the absolute bioavailability increased to 73% from 4% observed after oral selegiline administration (Azzaro et al. 2007). However, the high and sustained plasma concentration of selegiline after STS administration results in non-selective inhibition of MAO isoforms in the brain. In early clinical trials, high, probably non-selective, dose of selegiline was found to have antidepressant activity, but the risk of cheese effect hindered its use in this indication (Mann et al. 1989; Sunderland et al. 1994). It was also reported that even chronic daily oral treatment of parkinsonian patients with MAO-B inhibitors reduced plasma MAO-A activity by about 70% (Bartl et al. 2014), brain data, however, were not available. It was only recently demonstrated that about 33% inhibition of MAO-A was achieved in human brain after 28-day treatment with STS (6 mg/day) (Fowler et al. 2015). It is in accordance with the results of clinical trials showing its antidepressant effect. Since transdermal absorption avoids the gastrointestinal tract, no considerable intestinal MAO-A inhibition was expected and was first confirmed in an animal study (Mawhinney et al. 2003). Low risk of cheese effect was thus supposed.

Short-term placebo-controlled studies of 6 and 8 weeks in adult patients with major depressive disorder demonstrated statistically significant antidepressant activity of STS compared to placebo. The safety profile of the active compound was similar to that of placebo except for application-site reactions (Bodkin and Amsterdam 2002; Amsterdam 2003; Feiger et al. 2006). There was no hypertensive effect (cheese effect) observed with even consuming tyramine-enriched diet (Blob et al. 2007). The long-term effectiveness of STS was also demonstrated for prevention of relapse in those major depressive patients who initially responded to the preparation. In the STS group, less patients experienced relapse and the time till relapse was also longer compared to placebo (Amsterdam and Bodkin 2006). One 12-week study conducted on adolescents, however, failed to show its superiority to placebo likely due to the high placebo response rate (DelBello et al. 2014). STS has FDA approval from 2006 for the treatment of major depressive disorder in adults.

Transdermal selegiline was also studied for alleviation of nicotine and cocaine dependence. As it increases dopamine level in the brain, it was supposed it might reduce craving. Previously in a smaller trial adding oral selegiline to nicotine patch doubled the 52-week continuous abstinence rate, although the difference was not significant. The need of nicotine replacement in the selegiline group was mitigated, probably due to reduced craving (Biberman et al. 2003). However, in placebo-controlled trials STS failed to improve the effectiveness of cognitive behavior intervention in smoking abstinence (Killen et al. 2010; Kahn et al. 2012). In case of cocaine dependence, although preliminary data were promising the controlled clinical trial with STS also failed to show significant effect compared to placebo (Elkashef et al. 2006).

## Future perspectives

After more than half a century history of the first selective MAO-B inhibitor selegiline and its almost 40-year use in the treatment of PD, there is still a continuous interest in studying the unexplored activities of selegiline and other MAO inhibitors.

Promising cardio-metabolic effects of selegiline in animal studies were reported by several research groups. Selegiline showed antioxidant activity and reduced the fat accumulation in the liver of rats on lipid-rich diet (Bekesi et al. 2012). It also improved the metabolism and inflammation in adipose tissue of high-fat, high-sucrose diet-fed rats (Nagy et al. 2018). Its cytoprotective effect was shown in a rabbit model of chronic heart failure by reducing plasma norepinephrine, cardiac oxidative stress and myocyte apoptosis (Qin et al. 2003). MAO-B was also identified as a source of oxidative stress in the vasculature, especially in diabetes (Sturza et al. 2015). MAO-B inhibition by selegiline improved vascular function of human mammary arteries in patients with coronary heart disease regardless of the presence of diabetes (Lighezan et al. 2016). Alleviation of vascular hyperpermeability by selegiline was also demonstrated after hemorrhagic shock (Tharakan et al. 2010) and thermal injury (Whaley et al. 2009).

Potential anticancer activity of MAO inhibitors was also suggested. Overexpression of MAO in various cancer cells was reported and inhibition of the enzyme resulted in antiproliferative effect (Shih 2018; Gaur et al. 2019). Most data come from in vitro experiments, but phenelzine, a non-selective MAO inhibitor, is already in a phase II clinical trial for treatment of non-metastatic recurrent prostate cancer [ClinicalTrials.gov Identifier: NCT02217709], suggesting the clinical relevance of the anticancer activity of MAO inhibitors. High-dose selegiline itself was reported to exert cytotoxic effects on various cancer cell lines in vitro. Millimolar concentration of selegiline was shown to induce apoptotic cell death in melanoma cell line (Szende et al. 2000) and in acute myelogenous leukemia cells by inhibition of the mitochondrial respiration (Ryu et al. 2018). These very high concentrations, however, may suggest MAO-independent mechanisms that remain to be explored.

The several distinct pharmacological activities observed in in vitro and in animal experiments are really interesting, but still far from clinical relevance. The diversity of these effects may indicate either not yet characterized multiple functions of MAO enzyme protein or other targets of MAO inhibitor compounds.

## Abbreviations

ADL: Activity of daily living
MAO: Monoamine oxidase
ODT: Orally disintegrating tablets
PD: Parkinson’s disease
STS: Selegiline transdermal system
UPDRS: Unified Parkinson’s disease rating scale
